# Cone beam computed tomography in the assessment of TMJ deformity in children with JIA: repeatability of a novel scoring system

**DOI:** 10.1186/s12903-022-02701-5

**Published:** 2023-01-10

**Authors:** Thomas A. Augdal, Oskar W. Angenete, Xie-Qi Shi, Mats Säll, Johannes M. Fischer, Ellen Nordal, Karen Rosendahl

**Affiliations:** 1 Section of Paediatric Radiology, University Hospital of North Norway, Postboks 100, 9038 Tromsø, Norway; 2 Department of Clinical Medicine, Faculty of Health Sciences, UiT The Arctic University of Norway, Tromsø, Norway; 3 Department of Radiology and Nuclear Medicine, St. Olav University Hospital, Trondheim, Norway; 4 Faculty of Medicine and Health Sciences, Institute for Circulation and Medical Imaging, Norwegian University of Science and Technology, Trondheim, Norway; 5 Department of Clinical Dentistry, The Faculty of Medicine, University of Bergen, Bergen, Norway; 6 Department of Oral and Maxillofacial Radiology, Faculty of Odontology, University of Malmö, Malmö, Sweden; 7 Department of Paediatrics, University Hospital of North Norway, Tromsø, Norway

**Keywords:** Arthritis juvenile, Temporomandibular joint, Observer variation, Scoring system, Precision

## Abstract

**Background:**

The temporomandibular joint (TMJ) is frequently involved in juvenile idiopathic arthritis (JIA). Diagnostic imaging is necessary to correctly diagnose and evaluate TMJ involvement, however, hitherto little has been published on the accuracy of the applied scoring systems and measurements. The present study aims to investigate the precision of 20 imaging features and five measurements based on cone beam computed tomography (CBCT).

**Methods:**

Imaging and clinical data from 84 participants in the Norwegian study on juvenile idiopathic arthritis, the NorJIA study, were collected. Altogether 20 imaging features and five measurements were evaluated independently by three experienced radiologists for intra- and interobserver agreement. Agreement of categorical variables was assessed by Fleiss’, Cohen’s simple or weighted Kappa as appropriate. Agreement of continuous variables was assessed with 95% limits of agreement as advised by Bland and Altman.

**Results:**

“Overall impression of TMJ deformity” showed almost perfect intraobserver agreement with a kappa coefficient of 0.81 (95% CI 0.69–0.92), and substantial interobserver agreement (Fleiss’ kappa 0.70 (0.61–0.78)). Moreover, both “flattening” and “irregularities” of the eminence/fossa and condyle performed well, with intra- and interobserver agreements of 0.66–0.82 and 0.55–0.76, respectively. “Reduced condylar volume” and “continuity” of the fossa/eminence had moderate intra- and interobserver Kappa values, whereas continuity of the condyle had Kappa values above 0.55. Measurements of distances and angles had limits of agreement of more than 15% of the sample mean.

**Conclusions:**

We propose a CBCT-based scoring system of nine precise imaging features suggestive of TMJ deformity in JIA. Their clinical validity must be tested.

**Supplementary Information:**

The online version contains supplementary material available at 10.1186/s12903-022-02701-5.

## Background

Juvenile idiopathic arthritis (JIA) is an autoimmune condition, which includes all arthritides of unknown origin with onset before 16 years of age and duration more than six weeks [1]. JIA is the most common rheumatic disease of childhood with a prevalence of up to 1–2 per 1000 in developed countries [1].

Depending on the population examined, the method of ascertainment and the applied diagnostic criteria, the temporomandibular joint (TMJ) is involved in 39–78% of patients with JIA [2–6]. Approximately one-third of patients with JIA and TMJ arthritis will develop dentofacial deformities such as malocclusion, micro- or retrognathia and facial asymmetry, requiring dental care [7–9].

Both history and clinical findings of TMJ arthritis can be equivocal, emphasising the importance of imaging to detect and monitor active disease [10, 11]. Magnetic resonance imaging (MRI) is the preferred modality for evaluation of disk pathology and active TMJ arthritis, with visualisation of joint effusion and a thickened and hyperaemic synovium [12, 13]. The osseous structures, on the other hand, are better depicted with radiographic techniques. The tomographic nature of the panoramic technique is susceptible to image distortion, and can only be used as an overview to detect gross deformity [14]. Studies on dry skulls have shown that cone beam computed tomography (CBCT) has better sensitivity and similar reliability for detection of condylar pathology as compared to computed tomography (CT) [15, 16]. The effective dose varies greatly depending on the CBCT machine, field of view and applied exposure parameters. However, compared to CT, CBCT was reported to have about 35% lower radiation dose and better subjective image quality [17]. Newer MRI techniques have shown promising results in adults for assessment of TMJ shape, yet, taken together, osseous deformity is currently best assessed with CBCT [18].

The drawbacks with conventional radiography and the increasing importance of detecting early signs of TMJ involvement in JIA has led to increased interest in CBCT, both for diagnosis, follow-up and further research. However, to date, little has been published on the precision and reproducibility of features and measurements used for assessing TMJ deformity in children. For example studies used consensus instead of agreement [6, 19], gave incomplete information [20, 21], used inappropriate methods [22–24] or did not discuss agreement or precision at all [25–29]. This information is, however, key to a correct understanding and clinical use of the findings, as outlined in 1991 by Fryback and Thornbury in their widely cited paper [30].

The aims of the present study were to examine the precision and repeatability of a predefined set of 20 CBCT-based imaging features and five measurements used to describe TMJ deformity, and next to devise a scoring system based on the more robust features.

## Methods

The present study is part of the Norwegian JIA study (NorJIA), a prospective, longitudinal observational study performed between 2015 and 2020. Participants in the main study (n = 228) were recruited from three tertiary university hospitals in the Western, Central and Northern Norway Regional Health Authorities. Children aged 4–16 years were included if they met the diagnostic criteria of JIA according to the International League of Associations for Rheumatology (ILAR) Classification [31]. Written informed consent was obtained from all participants and/or their legal guardian/parent. As part of the study protocol all participants in the NorJIA study were referred for a CBCT. The exclusion criterion for the present study was suboptimal examination due to artefacts.

To examine the precision and repeatability of potential imaging features, a subset of CBCT examinations was selected from the NorJIA study population by one of the local radiologists (TAA, MS, XS) at each site. The selection was based on the CBCT report and demographic and clinical information to represent an a priori balanced range of imaging findings where approximately one-third had moderate/severe findings, one-third mild findings and one-third subtle or no findings. Given this prevalence, an expected Kappa coefficient of 0.6, and a precision of ± 0.15 at a confidence level of 90% estimated a sample size of 81 [32]. According to Bland a sample size of 100 for repeat measurements of continuous variables is good—giving 95% CIs about the upper and lower limits of agreement of approximately ± 0.34 × the standard deviation of the differences [33]. The completed checklist for the guidelines for reporting reliability and agreement studies (GRRAS) is found in Additional file 1 [34].

### Imaging

The CBCT examinations took place at the Regional Competence Centres for Oral Health. They were conducted by experienced radiographers with the participants positioned in the Frankfort plane horizontal with their teeth in maximal intercuspal position. CBCT model and machine settings are outlined in Table 1.

**Table 1:** CBCT machine (number of CBCT examinations in parenthesis), settings and DICOM viewers *(kVp, kilovoltage peak; mAs, milliampere-seconds; DICOM, digital imaging and communications in medicine)*

CBCT machine	kVp	mAs	Field of view (mm)	Isotropic voxel dimension (mm)	DICOM viewer
3D Accuitomo 170^a^ (n = 30)	85	175	40 × 40 × 40	0.08	iDixel One Volume viewer^a^
Promax 3D^b^ (n = 29)	90	13.6	200 × 200 × 60	0.40	Planmeca Romexis viewer^b^
Scanora 3D^c^ (n = 25)	90	45	60 × 60 × 60	0.13	OnDemand3DApp project viewer Limited^d^

^a^Morita MFG Corp, Kyoto, Japan

^b^Planmeca Oy, Helsinki, Finland

^c^Soredex, Tuusula, Finland

^d^CyberMed, Daejeon, Republic of Korea (version 1.0.10.4304)

### Image review

**Fig. 1:**
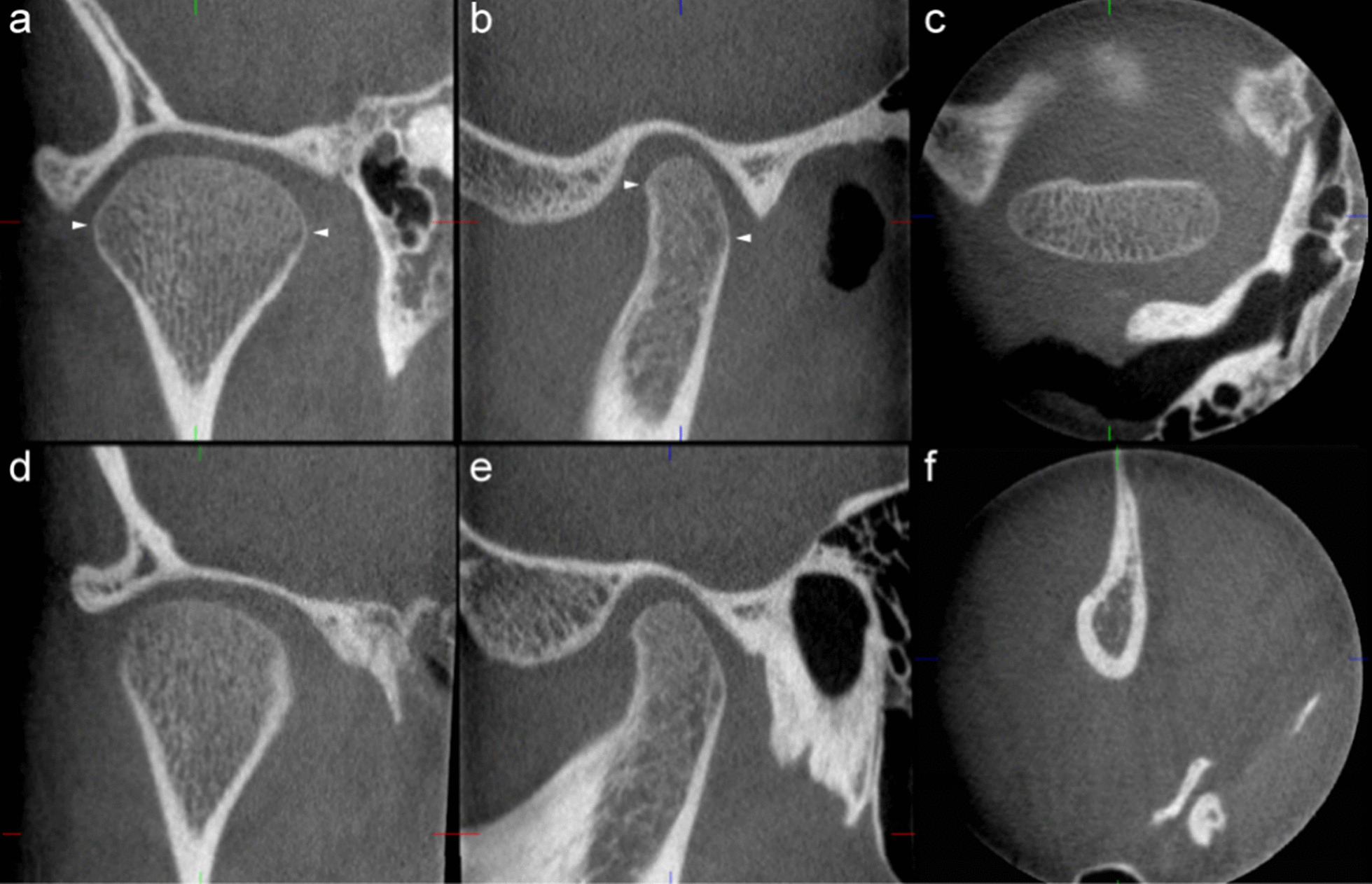
Image volume orientation models. Coronal (**a**, **d**), sagittal (**b**, **e**) and axial (**c**, **f**) view of the TMJ. **a**–**c** Condyle-corrected. In an axial view through the centre of the condyle, the sagittal plane is aligned perpendicular to the long axis (mesiolateral diameter) of the condyle. **d**–**f** Ramus-corrected. The sagittal plane is aligned from the coronoid process through the centre of the condyle in the axial view, and approximated to the longitudinal axis of the ramus in the coronal view. Arrowheads in **a** and **b** indicate the ‘equator’ in the variable ‘reduced condylar volume’

**Fig. 2:**
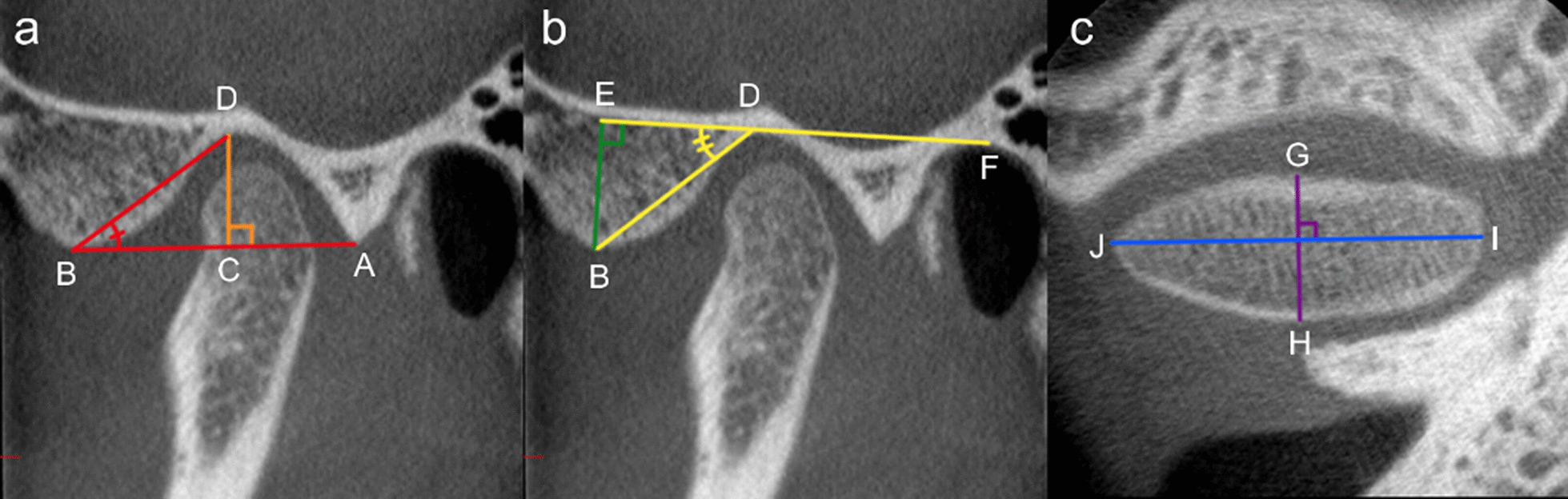
Linear and angular measurements of the glenoid fossa and condyle. Sagittal view of the glenoid fossa (**a**, **b**) and axial view of the condyle (**c**). **a** Method A. A reference line was drawn between the postglenoid process (A) and the apex of the articular eminence (B). Fossa depth (CD, orange) was measured from the deepest point of the fossa (D) to the reference line. Fossa length (AB, red) was measured along the reference line. The fossa-eminence inclination angle (ABD, red) was measured between the reference line and the deepest point of the fossa. **b** Method B. The depth of the glenoid fossa (BE, green) was measured from the apex of the articular eminence (B) to a horizontal line through the upper border of the external auditory canal (F) and the deepest point of the fossa (D). A fossa-eminence inclination angle (EDB, yellow) between the horizontal line and a line from the deepest point of the glenoid fossa to the apex of the articular eminence was constructed (according to reference [37]). **c** Anteroposterior (GH, violet) and mesiolateral (IJ, blue) diameter of the condyle

Prior to scoring calibration between readers was performed during a number of face-to-face and online meetings to address volume reorientation, identification of landmarks and multiplanar reconstructions. Further, imaging features and their grading were carefully discussed and standardised based on both single images and complete examinations with a particular focus on discrimination between categories. Based on previous literature five measurements (glenoid fossa depth and length, glenoid fossa/articular eminence inclination angle, and condyle length and width) and 20 imaging features describing anatomy and deformity (overall impression of TMJ deformity, condyle volume and position, joint surface continuity, irregularity and flattening of the condyle and glenoid fossa/articular eminence, apposition, ankylosis, heterotopic bone formation, loose joint body and findings suggestive of osteoarthritis) were identified for the present study [6, 26, 35–42]. Definitions of volume reorientation, measurements and variables are given in Figs. 1 and 2 and Table 2, respectively.

**Table 2:** CBCT imaging features for scoring of temporomandibular joint deformity in juvenile idiopathic arthritis

Imaging feature	Definition/explanation	Grading
Overall impression of TMJ deformity		0 = normal 1 = mild 2 = moderate/severe
Flattening of the articular eminence and glenoid fossa^a^	A change from the expected s-shaped appearance	0 = absent; i.e. s-shaped 1 = mild to moderate widening or flattening 2 = severely flattened fossa/eminence
Surface irregularity of the articular eminence and glenoid fossa^b^	Irregular change(s) of surface shape, for example depression. More distinct and sharply demarcated than ‘flattening’. May be continuous or discontinuous	0 = absent 1 = mild (involving only part of the articular surface, including multiple depressions/lesions) 2 = moderate/severe (involving the entire articular surface, or presence of deep brakes in the subchondral bone seen in two planes
Continuity of the articular eminence and glenoid fossa	The integrity of the articular (cortical) surface itself	0 = continuous 1 = discontinuous 2 = not applicable (due to sclerotic underlying bone)
Flattening of the condyle—sagittal view^b^	A flattening change from the expected rounded/ovoid shape	0 = absent, i.e. rounded/ovoid 1 = subtle anterior flattening 2 = mild flattening, involves part of the surface of the condyle 3 = Moderate/severe, involves the entire surface of the condyle, or loss of height of the condyle
Flattening of the condyle—coronal view	A flattening change from the expected convex shape	0 = absent, i.e. convex throughout 1 = mild or partial flattening 2 = moderately or severely flattened, or flattened throughout
Reduced condylar volume^c^	Generally reduced condylar volume. The condyle is defined cranial to the ‘equator’, i.e. the anterior, posterior, medial and lateral points of maximum convexity when the condyle and neck is viewed in the sagittal and coronal view, respectively	0 = normal 1 = mildly reduced volume/height (clearly above ‘equator’-level) 2 = moderately reduced volume/height (does not cross ‘equator’-level) 3 = severely reduced volume/height (‘equator’-level or lower, yet still fan shape in coronal view) 4 = cylinder shape in coronal view
Surface irregularity of the condyle^b^	Irregular change(s) of surface shape, for example depression. More distinct and sharply demarcated than ‘flattening’. May be continuous or discontinuous	0 = absent 1 = mild (involving only part of the articular surface, including multiple depressions/lesions) 2 = moderate/severe (involving the entire articular surface, or presence of deep brakes in the subchondral bone seen in two planes
Continuity of the condylar surface	The integrity of the articular (cortical) surface itself	0 = continuous 1 = discontinuous
Position of the condyle in the temporal fossa		0 = neutral 1 = anterior 2 = posterior 3 = medial 4 = lateral 5 = superior 6 = inferior
Subchondral sclerosis of the articular eminence and glenoid fossa^d^	A thickening of the cortical bone (load-bearing) and unequivocal involvement of the subchondral bone	0 = none 1 = thickened cortical bone 2 = subchondral sclerosis
Osteophyte of the articular eminence and glenoid fossa^e^	Marginal hypertrophy with sclerotic borders and exophytic angular formation of osseous tissue arising from the surface	0 = absent 1 = present
Subchondral pseudocyst of the articular eminence and glenoid fossa	A cyst-appearing cavity underlying the articular surface. To be discerned from variation of the trabecular bone	0 = absent 1 = present
Subchondral sclerosis of the condyle^d^	A thickening of the cortical bone (load-bearing) and unequivocal involvement of the subchondral bone	0 = none 1 = thickened cortical bone 2 = subchondral sclerosis
Osteophyte of the condyle^d^	Marginal hypertrophy with sclerotic borders and exophytic angular formation of osseous tissue arising from the surface	0 = absent 1 = present
Subchondral pseudocyst of the condyle	A cyst-appearing cavity underlying the articular surface. To be discerned from variation of the trabecular bone	0 = absent 1 = present
Bone apposition	Bony contact between the condyle and temporal bone	0 = absent 1 = present
Ankylosis	Continuous structure between the condyle and temporal bone	0 = absent 1 = fibrous (suspected) 2 = osseous
Loose joint body^d^	A well-defined calcified structure that is not continuous with the osseous structures of the joint	0 = absent 1 = present
Heterotopic calcification		0 = absent 1 = present

^a^Adapted from reference[41]

^b^Adapted from reference[39]

^c^Definition of condyle/’equator’ from reference[40]

^d^Adapted from reference[35]

^e^From reference[35]

The examinations were read independently by two radiologists and one dento-maxillofacial radiologist (TAA, OA and CX, with 13, 12 and 14 years of experience in imaging, respectively). The examinations were anonymised for all information except scan date and study site. Image viewing conditions were standardised (diagnostic screens, ambient lighting etc.) and the Digital Imaging and Communications in Medicine (DICOM) image viewers included in the three respective CBCT systems were used (details in Table 1). Adjustment of window-level settings was allowed. After an interval of minimum three weeks the reading was repeated by TAA.

To examine the potential of CBCT to specify the location of pathology, the glenoid fossa and the condyle were scored for irregularities and flattening by segmental scoring and as a whole, in separate sessions. However, preliminary results showed that segmental scoring had very poor agreement [43], and this was therefore omitted from further analysis.

### Statistics

Ordinal data are presented as medians (ranges), dichotomous data as proportions and continuous data as medians (IQR) or means (±SD). For categorical variables, intra- and interobserver agreement was assessed with kappa (κ) coefficients (95% confidence interval). Fleiss’ kappa was applied for three observers and Cohen’s simple or linear weighted kappa for two observers or observations. A κ coefficient of < 0 was considered poor, 0–0.20 slight, 0.21–0.40 fair, 0.41–0.60 moderate, 0.61–0.80 substantial and 0.81–1.00 almost perfect [44]. For continuous variables, intra- and interobserver agreement was assessed by calculating the mean difference and standard deviation (SD) of the differences to establish the 95% limits of agreement (LOA, mean difference ± 1.96*SD) as advised by Bland and Altman [45]. Outliers were removed from final analysis if the value was more than four standard deviations from the mean [46]. Bland–Altman plots are usually informally interpreted, and we set the limit for clinically acceptable agreement (100: sample mean × 95% LOA) at 15%. The mean differences were used as a measure of bias, and considered statistically significant given a p-value < 0.05 (two tailed, one-sample t-test). Proportional bias was assumed if linear regression was statistically significant. To assess the potential impact of different CBCT-systems, intraobserver analyses were repeated stratified by study site for the categorical variables, and a one-way between-groups analysis of variance was conducted for the intraobserver mean differences. All statistical analysis was performed using IBM SPSS version 28 (IBM, Chicago, IL). The level of statistical significance was set at 5% (p-value < 0.05).

## Results

84 children (51 girls) with JIA were included (84 CBCT examinations). Median age at CBCT examination was 14.3 years (IQR 4.3), median age at diagnosis was 6.2 years (IQR 8.7) and median disease duration at the time of the CBCT was 6.3 years (IQR 6.3) (Table 3).

**Table 3:** Characteristics of 84 children with a known diagnosis of JIA, included in the present study (ILAR, International League of Associations for Rheumatology; IQR, inter-quartile range; JIA, juvenile idiopathic arthritis; CBCT, cone beam computed tomography)

Characteristics	Values
Girls, n (%)	51 (61)
Age at CBCT examination, median years (IQR)	14.3 (11.5–15.7)
Age at JIA diagnosis, median years (IQR)	6.2 (2.3–11.0)
Disease duration, median years (IQR)	6.3 (3.8–10.0)
*JIA categories, n (%)*	
Systemic	3 (4)
Oligoarticular persistent	27 (32)
Oligoarticular extended	10 (12)
Polyarticular Rheumatoid Factor positive	1 (1)
Polyarticular Rheumatoid Factor negative	24 (29)
Psoriatic arthritis	2 (2)
Enthesitis-related arthritis	9 (11)
Undifferentiated arthritis	8 (10)

### Imaging features

**Fig. 3:**
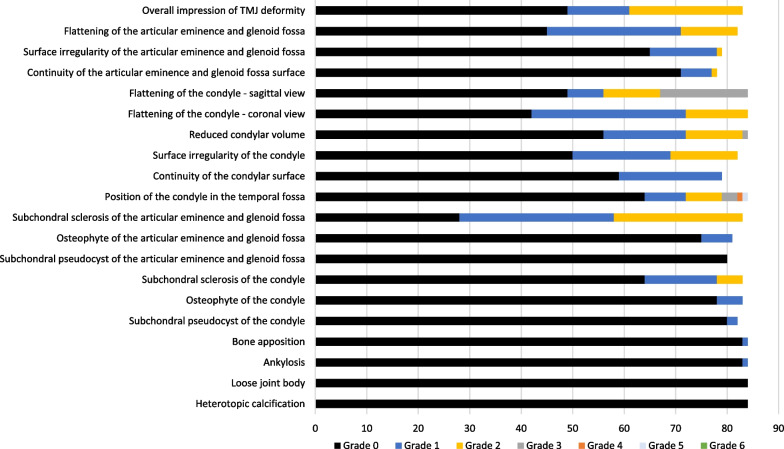
Distribution of findings, right side, 1st reading. The x-axis denotes number of participants

**Fig. 4:**
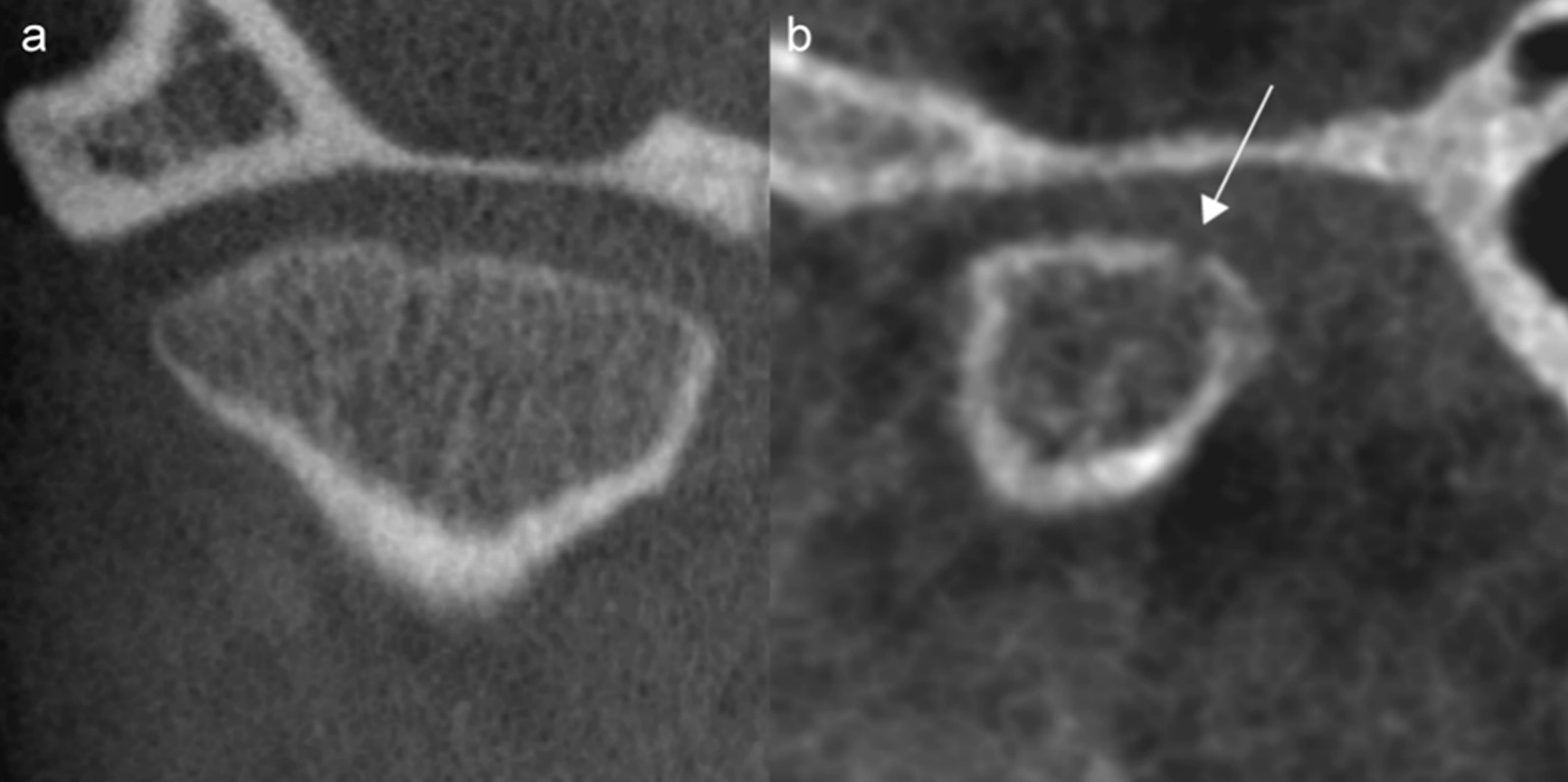
Grading of continuity of the articular surface. Coronal (**a**) and sagittal (**b**) view of the TMJ. **a** Grade 0, continuous outline of the glenoid fossa and condyle, the discrete condylar irregularity is continuous. **b** Grade 1, a discontinuity (arrow) posteriorly in the condyle

**Fig. 5:**
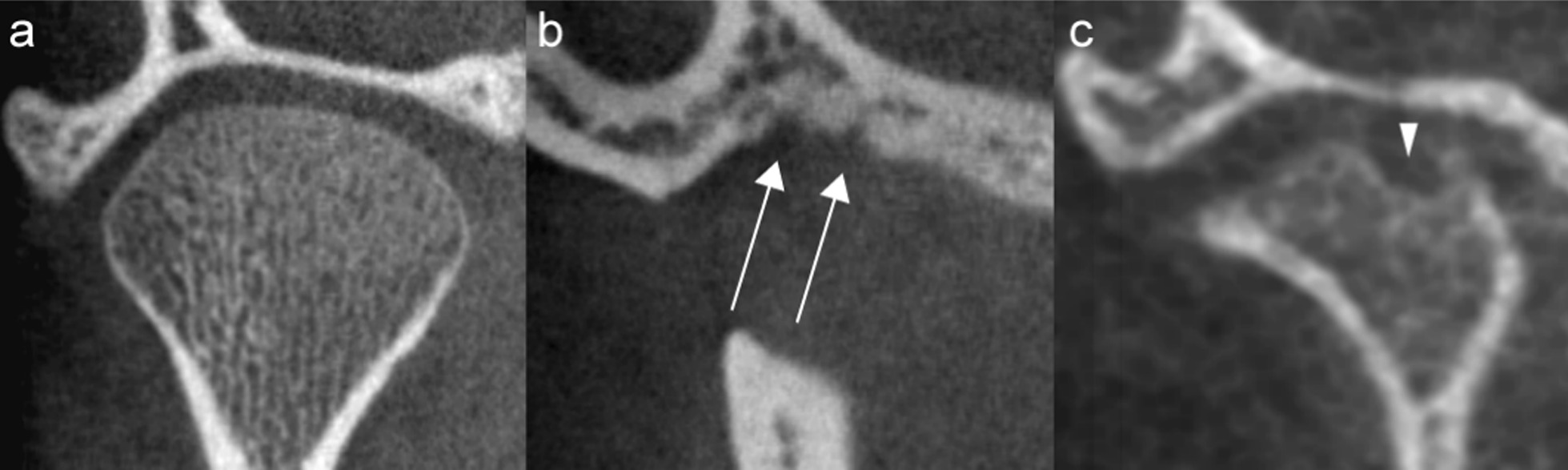
Grading of irregularities. Coronal views of the TMJ. **a** Grade 0, smooth outline of the glenoid fossa and the condyle. **b** Grade 1, mild irregularity. Depressions (arrows) in the central part of the glenoid fossa. **c** Grade 2, moderate/severe irregularity. Deep brake (arrowhead) in the condyle

**Fig. 6:**
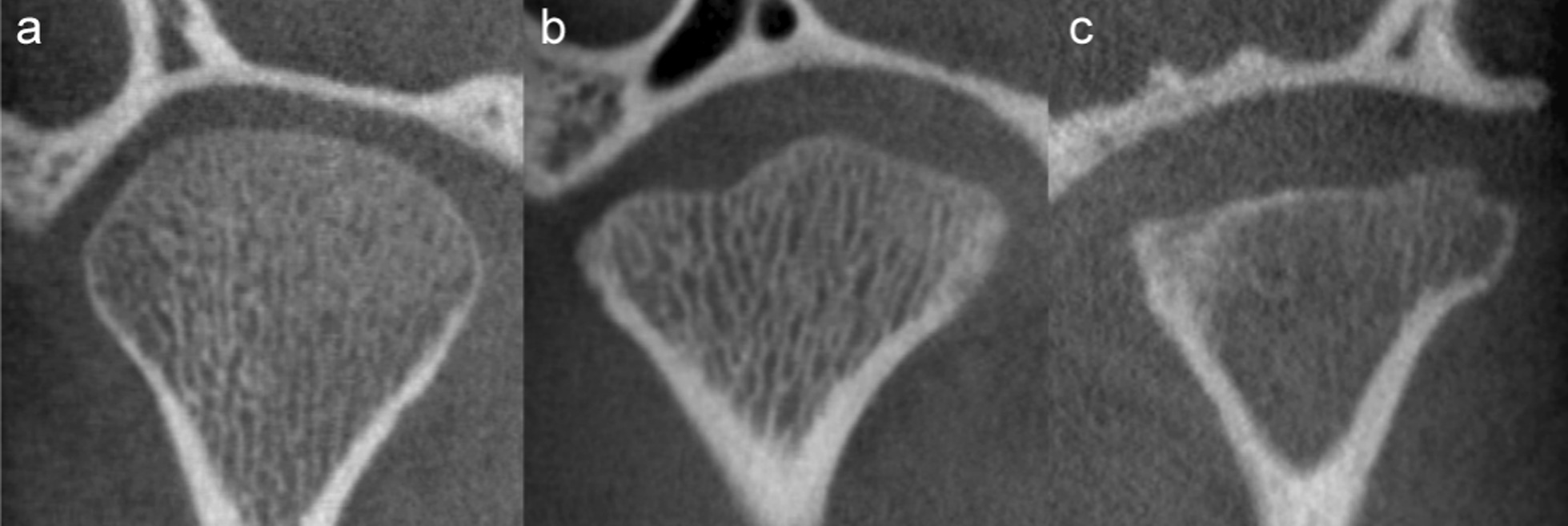
Flattening of the condyle in the coronal view. **a** Grade 0, absent, i.e. convex throughout. **b** Grade 1, mild or partial flattening. **c** Grade 2, moderately or severely flattened, or flattened throughout

**Fig. 7:**
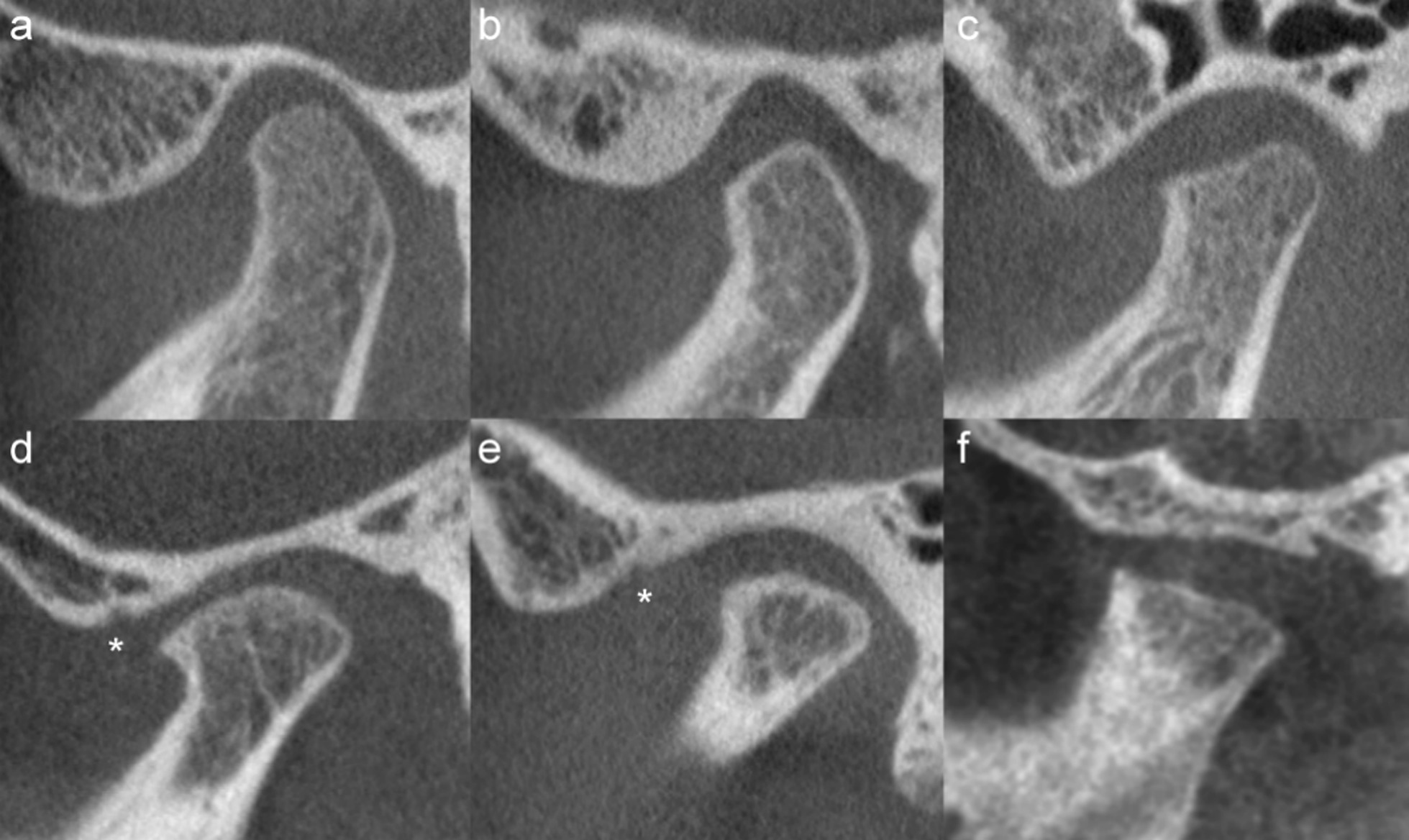
Flattening of the articular eminence and glenoid fossa and condyle in the sagittal view. **a** Both Grade 0. **b** Fossa grade 0. Condyle grade 1, subtle anterior flattening. **c** Fossa grade 1, mild widening or flattening. Condyle grade 2, mild flattening, involves part of the surface of the condyle. **d** Fossa grade 1 mild to moderate widening or flattening. Condyle grade 3, moderate flattening, loss of height of the condyle. **e** Fossa grade 1, moderate widening or flattening. Condyle grade 3, moderate flattening and loss of height of the condyle. **f** Fossa grade 2, severely flattened fossa/eminence. Condyle grade 3, severe flattening, involves the entire surface of the condyle and loss of height of the condyle. Note also the irregularities (asterisk) in the articular eminence/glenoid fossa in (**d**) and (**e**), the osteophyte at the anterior part of the condyle in (**d**) and the thickened, sclerotic appearance of the condyle in (**e**)

The distribution of scores for each of the CBCT-features are given in Fig. 3 (right TMJ, first reading). Six features were not further analysed due to severely skewed distribution. Absolute, intra- and interobserver agreement for the remaining 14 features are detailed in Table 4. Examples of continuity of the articular surface, irregularities and flattening are shown in Figs. 4, 5, 6, and 7.

**Table 4:** Intra- and interobserver kappa coefficients (95% confidence interval) and interobserver absolute agreement (%) for CBCT imaging features describing anatomy and deformity in a sample of 84 children with JIA, right TMJ. Cohen's linear weighted kappa coefficients unless specified. Grading outlined in Table 2

Imaging feature	Intra-observer kappa coefficient	Interobserver kappa coefficient	Interobserver absolute agreement (%)
Overall impression of TMJ deformity (0–2)	0.81 (0.69–0.92)^a^	0.70 (0.61–0.79)^b^	64/82 (78%)
Flattening of the articular eminence and glenoid fossa (0–2)	0.77 (0.66–0.89)	0.65 (0.51–0.79)	62/82 (76%)
Surface irregularity of the articular eminence and glenoid fossa (0–2)	0.66 (0.48–0.84)	0.55 (0.32–0.79)	70/79 (88%)
Continuity of the articular eminence and glenoid fossa surface (0–2)	0.46 (0.09–0.82)^a^	0.43 (0.30–0.56)^b^	67/76 (88%)
Flattening of the condyle—sagittal view (0–3)	0.82 (0.75–0.90)	0.76 (0.67–0.85)	59/83 (71%)
Flattening of the condyle—coronal view (0–2)	0.71 (0.58–0.83)	0.60 (0.46–0.74)	60/84 (71%)
Reduced condylar volume (0–4)	0.58 (0.43–0.73)^a^	0.47 (0.38–0.56)^b^	50/83 (60%)
Surface irregularity of the condyle (0–2)	0.77 (0.66–0.89)	0.70 (0.57–0.83)	65/82 (79%)
Continuity of the condylar surface (0–1)	0.66 (0.46–0.85)^a^	0.51 (0.38–0.63)^b^	55/77 (71%)
Position of the condyle in the glenoid fossa (0–6)	0.38 (0.18–0.58)^a^	0.10 (0.02–0.18)^b^	32/83 (39%)
Subchondral sclerosis in the articular eminence and glenoid fossa (0–2)	0.65 (0.52–0.78)	0.60 (0.47–0.73)	51/79 (65%)
Osteophyte of the articular eminence and glenoid fossa (0–1)	0.64 (0.31–0.97)^a^	0.46 (0.09–0.83)^a^	73/79 (92%)
Subchondral sclerosis of the condyle (0–2)	0.78 (0.64–0.92)	0.63 (0.46–0.81)	70/82 (85%)
Osteophyte of the condyle (0–1)	0.65 (0.28–1.00)^a^	0.37 (0.10–0.64)^a^	71/82 (85%)

^a^Cohen’s simple kappa

^b^Fleiss’ kappa

Assessment of the overall impression of TMJ deformity on a 0–2 scale showed almost perfect agreement for the same reader, with a kappa coefficient of 0.81 (95% CI 0.69–0.92) (Table 4). The interobserver agreement was substantial, with a Fleiss’ kappa coefficient of 0.70 (0.61–0.78). The absolute agreement between three observers was 64 out of 82 (78%) (Table 4).

There was a substantial to moderate agreement for the assessment of flattening of the articular eminence and glenoid fossa, surface irregularity and continuity of the articular eminence and glenoid fossa on a 0–2 scale, with kappa coefficients of 0.77, 0.66 and 0.46, respectively, for the same reader. The inter-reader agreement was moderate to substantial with kappa coefficients between 0.43 and 0.65 (Table 4).

Assessment of condylar flattening, both on a sagittal view, 0–3 scale, and on a coronal view, 0–2 scale, showed almost perfect and substantial agreement for the same reader, with kappa coefficients of 0.82 and 0.71, respectively. The inter-reader agreement was substantial and moderate, with kappa coefficients of 0.76 and 0.60 (Table 4). There was a substantial to moderate intra- and inter-reader agreement for the assessment of condylar surface irregularity, reduced condylar volume and continuity of the condyle, as well as for assessment of secondary degenerative change such as subchondral sclerosis and the presence of osteophytes (Table 4). For assessment of condyle position within the glenoid fossa the interobserver agreement was slight, k = 0.10, and intra-observer agreement fair, k = 0.38. Analysis stratified for study site did not change the results.

### Measurements

**Fig. 8:**
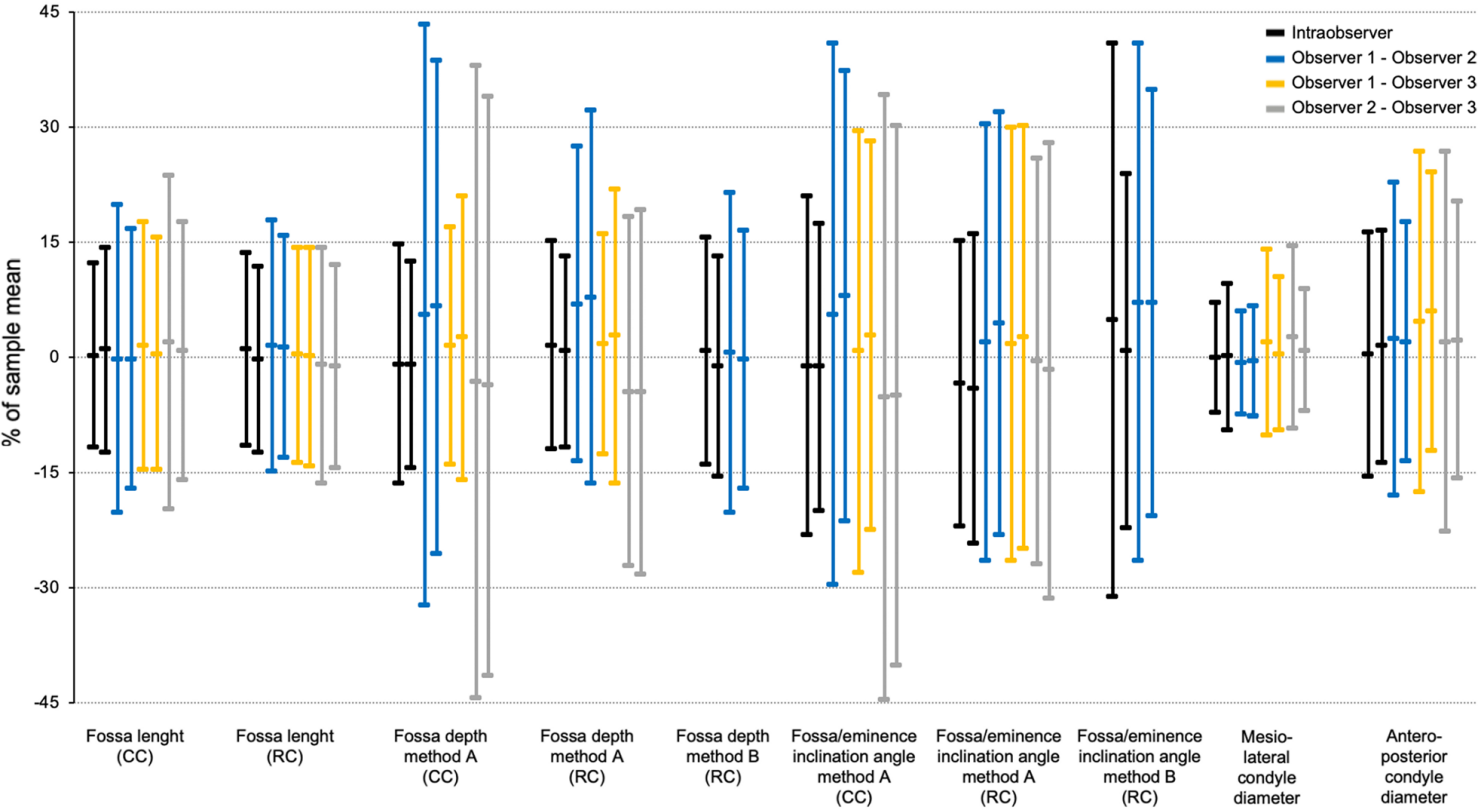
Variability of TMJ measurements. Each line represents the mean difference in percentage of the mean value (mean difference/mean × 100%) with the corresponding 95% limits of agreement in percent [(mean difference/mean × 100%) ± (1.96 SD/mean × 100%)]. Abbreviations: CC, condyle-corrected; L, left; R, right; RC, ramus-corrected

Measurements and differences resembled normal distribution patterns. The mean glenoid fossa lengths and depths, mean glenoid fossa/articular eminence inclination angles and the mean condylar diameters are given in Table 5. In the final analysis 24 outliers were removed. Bland–Altman plots of differences in all measurements showed relatively wide 95% limits of agreement, varying from 13.6 to 83.8% of the sample means (Table 5). Measurement variation both within and between the observers is illustrated in Fig. 8.

**Table 5:** Intra- and interobserver variability of TMJ measurements. Observer mean (SD), intra- and interobserver mean difference (SD) in a sample of 84 children with JIA. Distances in millimetre. Definitons of image volume orientation model and measurements given in Figs. 1 and 2*(CC, condyle-corrected; RC, ramus-corrected, Diff, difference; L, left; R, right; Obs, observer)*

	Image volume orientation model	Side	Observer 1		Intra-observer	Observer 2	Observer 3	Inter-observer		
								Obs1-Obs2	Obs1-Obs3	Obs2-Obs3
			1st mean (SD)	2nd mean (SD)	Mean diff (SD)	Mean (SD)	Mean (SD)	Mean diff (SD)	Mean diff (SD)	Mean diff (SD)
Fossa length	CC	R	16.7 (1.8)	16.7 (1.8)	0.0 (1.0)	16.8 (1.9)	16.5 (2.1)	0.0 (1.7)	0.2 (1.4)	0.3 (1.8)
		L	16.7 (2.0)	16.6 (1.9)	0.2 (1.1)	16.7 (1.9)	16.6 (2.1)	0.0 (1.4)	0.1 (1.3)	0.1 (1.4)
Fossa length	RC	R	17.1 (1.9)	17.0 (1.9)	0.2 (1.1)	16.9 (1.9)	17.0 (2.1)	0.3 (1.4)	0.0 (1.2)	− 0.2 (1.3)
		L	17.1 (1.9)	17.0 (2.0)	0.0 (1.1)	16.8 (2.0)	17.0 (2.0)	0.2 (1.2)	0.0 (1.2)	− 0.2 (1.1)
Fossa depth, method A	CC	R	5.7 (1.5)	5.7 (1.5)	− 0.1 (0.5)	5.5 (1.1)	5.5 (1.5)	0.3* (1.1)	0.1* (0.4)	− 0.2 (1.2)
		L	5.6 (1.1)	5.7 (1.1)	− 0.1 (0.4)	5.3 (1.1)	5.5 (1.2)	0.4* (0.9)	0.1 (0.5)	− 0.2 (1.0)
Fossa depth, method A	RC	R	5.6 (1.4)	5.5 (1.4)	0.1* (0.4)	5.3 (1.3)	5.5 (1.4)	0.4* (0.6)	0.1* (0.4)	− 0.2* (0.6)
		L	5.6 (1.0)	5.6 (1.8)	0.0 (0.4)	5.1 (1.0)	5.4 (1.1)	0.4* (0.7)	0.2* (0.5)	− 0.2* (0.6)
Fossa depth, method B	RC	R	6.7 (2.2)	6.6 (2.1)	0.1 (0.5)	6.6 (2.2)		0.0 (0.7)		
		L	6.6 (1.7)	7.0 (3.8)	− 0.1 (0.5)	6.6 (1.8)		0.0 (0.6)		
Fossa/eminence inclination angle, method A	CC	R	32.1 (8.5)	32.5 (7.7)	− 0.4 (3.6)	31.1 (6.1)	32.1 (9.3)	1.8* (5.7)	0.2 (4.7)	− 1.7* (6.4)
		L	32.4 (6.4)	32.8 (6.2)	− 0.4 (3.1)	30.0 (5.7)	31.6 (7.1)	2.5* (4.7)	0.9 (4.1)	− 1.5* (5.5)
Fossa/eminence inclination angle, method A	RC	R	30.6 (8.0)	32.2 (8.2)	− 1.1* (3.0)	30.6 (7.4)	30.8 (8.8)	0.6 (4.4)	0.5 (4.4)	− 0.2 (4.1)
		L	31.1 (6.7)	32.2 (6.4)	− 1.3* (3.3)	29.8 (5.8)	30.3 (7.6)	1.3* (4.3)	0.8 (4.3)	− 0.5 (4.5)
Fossa/eminence inclination angle, method B	RC	R	37.3 (9.4)	35.6 (10.4)	1.8* (6.7)	34.4 (10.9)		2.6* (6.2)		
		L	36.6 (8.9)	36.1 (9.4)	0.3 (4.3)	33.9 (9.6)		2.5* (5.0)		
Mesio-lateral condyle diameter	CC	R	16.5 (2.8)	16.6 (2.6)	0.0 (0.6)	16.7 (2.7)	16.3 (3.0)	− 0.1 (0.6)	0.3* (1.0)	0.4* (1.0)
		L	16.7 (2.3)	16.7 (2.5)	0.0 (0.8)	16.8 (2.3)	16.5 (2.8)	− 0.1 (0.6)	0.1 (0.8)	0.1 (0.7)
Anteroposterior condyle diameter	CC	R	7.5 (1.2)	7.5 (1.1)	0.0 (0.6)	7.3 (1.3)	7.2 (1.3)	0.2* (0.8)	0.3* (0.8)	0.1 (0.9)
		L	7.5 (1.2)	7.5 (1.4)	0.1 (0.6)	7.4 (1.4)	7.2 (1.4)	0.1* (0.6)	0.4* (0.7)	0.2* (0.7)

*p < 0.05

The mean differences (bias) for the same, and between observers ranged from 0.0 to 0.2 mm and from 0.0 to 0.4 mm for linear measurements. For the angular measurements the corresponding figures were 0.3–1.8 and 0.2–2.6 degrees. The mean differences were significantly different from zero (p < 0.05) in a number of measurements, as noted in Table 5. Furthermore, there was proportional bias in 17 out of 72 measurement pairs, of which 12 had a positive slope. Examples of Bland–Altman plots without and with bias are given in Additional file 2. In the stratified analysis the mean differences were not significantly different between the study sites in 19 out of 20 measurements.

## Discussion

In this study we have identified a set of nine robust CBCT-based image markers suggestive of TMJ deformity in children and adolescents with JIA. These include an overall impression of TMJ deformity, subjective assessment of condylar volume, joint surface continuity, surface irregularity and flattening of the condyle and articular eminence and glenoid fossa. Their clinical validity remains to be determined. Importantly, we also found that measurements of distances and angles performed poorly with wide limits of agreement.

We have shown that the overall impression of TMJ deformity can be reliably scored on CBCT, both for the same and between observers when using the three categories normal, mild or moderate/severe deformity. Our results compare well with those of Stoustrup and colleagues, who, in a study of 47 JIA patients and 19 non-JIA patients examined with a large field of view CBCT to assess associations between condylar changes and facial asymmetry, re-assessed 20 randomly selected patients to examine intra-observer agreement [42]. They found a substantial agreement for both discrimination of normal from pathological condyles and categorization of the pathological condyles as predominately deformed or eroded, with kappa values of 0.67 and 0.63, respectively. The same group later added a fourth category (combined deformation plus erosion) to their scoring system and reported an almost perfect intra-observer agreement (kappa = 0.83) for reassessment of 30 of 245 CBCTs in a long-term follow up examination of the Nordic JIA-cohort [4]. Our approach differed in that we assessed surface irregularities, flattening and signs of osteoarthritis separately, for both the glenoid fossa/articular eminence and for the condyle. We used up to four categories, thus allowing for a more detailed evaluation. All assessments performed well, both for the same and between observers.

Moreover our suggested scoring system allows for evaluation of the continuity of the joint surfaces. Importantly, this continuity, or loss of continuity, can be accurately distinguished from irregularities—which we defined as changes of shape more sharply demarcated than flattening, that may be continuous or not. According to the RDC/TMD-criteria [35] a surface erosion is defined as loss of continuity. However, this definition does not correspond well with the provided image examples, where irregularities/bony depressions are shown rather than loss of surface continuity. A distinction similar to ours was most likely included by Arvidsson et al. [6] as “cortical defect with/without sclerotic border”, but to the best of our knowledge its agreement has not been previously examined. We speculate that this marker might represent early involvement, not visualised on MRI. This is of interest as it may allow more precise and early monitoring of treatment response. The somewhat less favourable agreement for assessing the temporal versus the mandibular component of the joint might be due to the lower proportion of pathological findings in the glenoid fossa/articular eminence.

Following a consensus process and reliability exercise for three previously suggested MRI-scoring systems, Tolend and colleagues suggested 3-point scales for erosions and for condylar flattening in the oblique sagittal plane [39]. The scales tested in their reliability exercises for these two items were different from the final suggestion. However, in both systems both items met their predefined threshold for acceptable reliability, i.e. average measure intraclass correlation > 0.80 and smallest detectable difference < 30%, also suggesting robustness, yet not directly comparable to kappa coefficients.

We have previously suggested that condylar flattening as assessed from the coronal plane is a robust imaging feature [36]. In the present study on JIA patients, flattening of the condyle could be accurately scored from both the coronal and sagittal views. We acknowledge that a flattened articular eminence/glenoid fossa as a consequence of JIA was suggested decades ago, and has more recently been added to an MRI scoring system, however, this features’ agreement has not been examined previously [6, 37, 41, 47].

Condylar volume can be quantified with semiautomated techniques [48]. However, these techniques are more time consuming than subjective assessment. To the best of our knowledge, our study is the first to examine the agreement of subjective grading of reduced condylar volume. Scoring on a 0–4 scale performed well for the same reader, whilst the agreement between three assessors was fair, but still appropriate for clinical use. Perhaps not comparable with grading of pathology, yet relevant for classification of appearance, such as reduced condylar volume, Karlo and co-workers reported substantial interobserver agreement, k = 0.67, for classification of the condyle into one of three types in 210 children examined with CT for reasons not related to rheumatic or TMJ-disease [38].

The RDC/TMD original paper did not report agreement for each of their suggested research diagnostic criteria, but stated substantial agreement (kappa coefficient 0.71, absolute agreement 86%) between three observers for a dichotomous diagnosis of osteoarthritis in 145 joints in adults examined with CT [35]. We did not assess for osteoarthritis as such, but found similar agreement for its acknowledged separate elements subchondral sclerosis and osteophytes. Subchondral cysts and osteophytes occurred rarely, thus weakening our results, and we therefore suggest that osteoarthritis is scored dichotomized in accordance with the RCD/TMD criteria [35].

Ikeda and Kawamura reported an association between disc displacement and the position of the condyle within the glenoid fossa based on measurements of the joint space [49]. We found that subjective assessment of condylar position within the glenoid fossa on a 7-position scale performed poorly and is not sufficiently accurate for clinical use. Moreover, contrary to Ikeda and Kawamura, we found measurements of angles and small structures too imprecise for clinical use, with wide variation within and between observers and LOA outside our predefined limit of 15% of the sample means.

Our results are in line with those of Kellenberger et al. [37], in a MR study of 18 adolescents with anterior disc displacement and 18 patients with JIA. They found, based on a subset of 12 TMJs in 6 patients, a mean difference of 0.05 mm between two observers measuring the glenoid fossa depth, with wide limits of agreement (LOA -1.35 to 1.46) corresponding to 55% of the sample mean. Corresponding figures for the glenoid fossa/articular eminence inclination angle were 1.5 degrees and 55% of the sample mean, respectively. Similarly, Karlo and colleagues retrospectively measured the anteroposterior and mesiolateral diameter of the condyles on CT examinations in 210 children [38]. They reported wide interobserver LOA for both mesiolateral (− 2.8 to 2.0 mm) and anteroposterior diameter (− 2.0 to 1.6 mm), or 49% and 34% of the respective sample means. We believe that a substantial part of the observed variation is a reflection of the inherent variability in patient positioning, scan orientation during acquisition or volume reorientation prior to image review, in addition to the difficulties in defining the exact measurement points, as also noted by others [50].

Given our results, we suggest a novel, CBCT based scoring system for future studies based on the most robust features identified (Table 6). To the best of our knowledge, this is the first comprehensive scoring system that has been established for CBCT in the assessment of TMJ pathology in JIA.

**Table 6:** Proposed scoring system for CBCT-based evaluation of TMJ deformity in children with JIA

CBCT imaging feature	Grading
Overall impression of deformity	0–2
Flattening of the articular eminence and glenoid fossa	0–2
Flattening of the condyle—sagittal view	0–3
Flattening of the condyle—coronal view	0–2
Reduced condylar volume	0–4
Surface irregularity^a^	0–2
Continuity of the joint surface^a^	0–1
Osteoarthritis^b^	0–1

^a^Separate evaluation for articular eminence/glenoid fossa and condyl

^b^From Reference[35]

### Limitations and strengths

There are limitations to our study. Firstly, there is the subjective nature of developing any scoring system with inherent biases in readers understanding of what to score. To overcome this, several calibration meetings were held and an atlas depicting examples with different scores was established. Secondly, we were unable to include CBCT of healthy volunteers for ethical reasons. Of note approximately half the joints were scored with no or minor pathology. Thirdly, there are limitations to the use of Cohen’s kappa, particularly in datasets with skewed distributions [51]. We therefore present the proportion agreement and the distribution of scores to increase the transparency of the results. Fourthly, we chose to use only two or three observers to assess the scoring system’s potential given optimal conditions, rather than in a clinical setting. Fifthly, we acknowledge that despite efforts to obtain a balanced material, we were unable to properly assess the precision of rarely occurring imaging features of JIA, like subchondral pseudocyst, osteophytes, ankylosis, apposition, heterotopic calcification and loose joint body, due to their very low prevalence and even absence in our material. Finally, the CBCT machines were from different vendors between the sites, and hence the scan parameters had to be adapted locally. The differences in spatial resolution could potentially have influenced the ability to discriminate/detect discrete pathological findings and landmarks, however, this issue was thoroughly addressed during the calibration sessions and stratified analysis showed no relevant differences between study sites.

The strengths of this study include a large sample of children and adolescents with JIA, a meticulous standardization including construction of an atlas, the examination of both precision and repeatability and the use of image volumes instead of single images or stacks of images, thus reflecting the entire process of reading images. We included a breadth of pathology and severity of imaging features which were assessed by a scoring system that tested a wide variety of CBCT features of JIA.

## Conclusions

We have identified a set of robust CBCT-features suggestive of TMJ deformity in children with JIA, upon which a novel scoring system is proposed.

## Supplementary Information

**Additional file 1.** The completed checklist for the guidelines for reporting reliability and agreement studies (GRRAS).

**Additional file 2.** Examples of Bland–Altman plots without and with bias.

## Data Availability

The datasets used and/or analysed during the current study are available from the corresponding author on reasonable request.

## Abbreviations

CBCT: Cone beam computed tomography
CT: Computed tomography
DICOM: Digital imaging and communications in medicine
ILAR: International League of Associations for Rheumatology
IQR: Interquartile range
JIA: Juvenile idiopathic arthritis
LOA: Limits of agreement
MRI: Magnetic resonance imaging
RDC/TMD: Research diagnostic criteria for temporomandibular disorders
SD: Standard deviation
TMJ: Temporomandibular joint
