# Supplementary material for: Cone beam computed tomography in the assessment of TMJ deformity in children with JIA: repeatability of a novel scoring system
Source: BMC Oral Health. 2023 Jan 10;23:12. doi: 10.1186/s12903-022-02701-5 (PMC9830735; doi:10.1186/s12903-022-02701-5)
Supplement: Supplementary file 1 — Additional file 1. The completed checklist for the guidelines for reporting reliability and agreement studies (GRRAS). [file 12903_2022_2701_MOESM1_ESM.docx]

Additional file 1. **Guidelines for reporting reliability and agreement studies (GRRAS) checklist.** Version based on Table I in reference 34.

| **Section** | **Item**  **#** | **Checklist item** | **Reported**  **on page #** |
| --- | --- | --- | --- |
| Title/abstract | 1 | Identify in title or abstract that interrater/intrarater  reliability or agreement was investigated. | 1 |
|  | 2 | Name and describe the diagnostic or measurement  device or interest explicitly. | 4 |
|  | 3 | Specify the subject population of interest. | 3 |
|  | 4 | Specify the rater population of interest (if applicable). |  |
|  | 5 | Describe what is already known about reliability and  agreement and provide a rationale for the study (if  applicable). | 3-4 |
| Methods | 6 | Explain how the sample size was chosen. State the  determined number of raters, subjects/objects, and  replicate observations. | 5 |
|  | 7 | Describe the sampling method. | 5 |
|  | 8 | Describe the measurement/rating process (e.g. time  interval between repeated measurements, availability  of clinical information, blinding). | 6-7 |
|  | 9 | State whether measurements/ratings were conducted  independently. | 6 |
|  | 10 | Describe the statistical analysis. | 7 |
| Results | 11 | State the actual number of raters and subjects/objects  which were included and the number of replicate  observations which were conducted. | 8 |
|  | 12 | Describe the sample characteristics of raters and  subjects (e.g. training, experience). | 6 |
|  | 13 | Report estimates of reliability and agreement including  measures of statistical uncertainty. | 8-10 |
| Discussion | 14 | Discuss the practical relevance of results. | 10-14 |
| Auxiliary  material | 15 | Provide detailed results if possible (e.g. online). |  |
