# Supplementary material for: Cone beam computed tomography in the assessment of TMJ deformity in children with JIA: repeatability of a novel scoring system
Source: BMC Oral Health. 2023 Jan 10;23:12. doi: 10.1186/s12903-022-02701-5 (PMC9830735; doi:10.1186/s12903-022-02701-5)

Additional file 2. **Examples of Bland-Altman mean-difference plot.** X-axis denotes the mean fossa-eminence inclination angle, method A, condyle-corrected. Y-axis denotes the intra- or interobserver difference. Mean difference (red line a-d), upper and lower 95% limits of agreement (green lines a-d), and regression line (black c, d). (a) No bias or proportional bias, intraobserver left side. (b) Proportional bias, intraobserver right side. (c) Bias, interobserver 1-2, left side. (d) Bias and proportional bias, interobserver 2-3, right side.


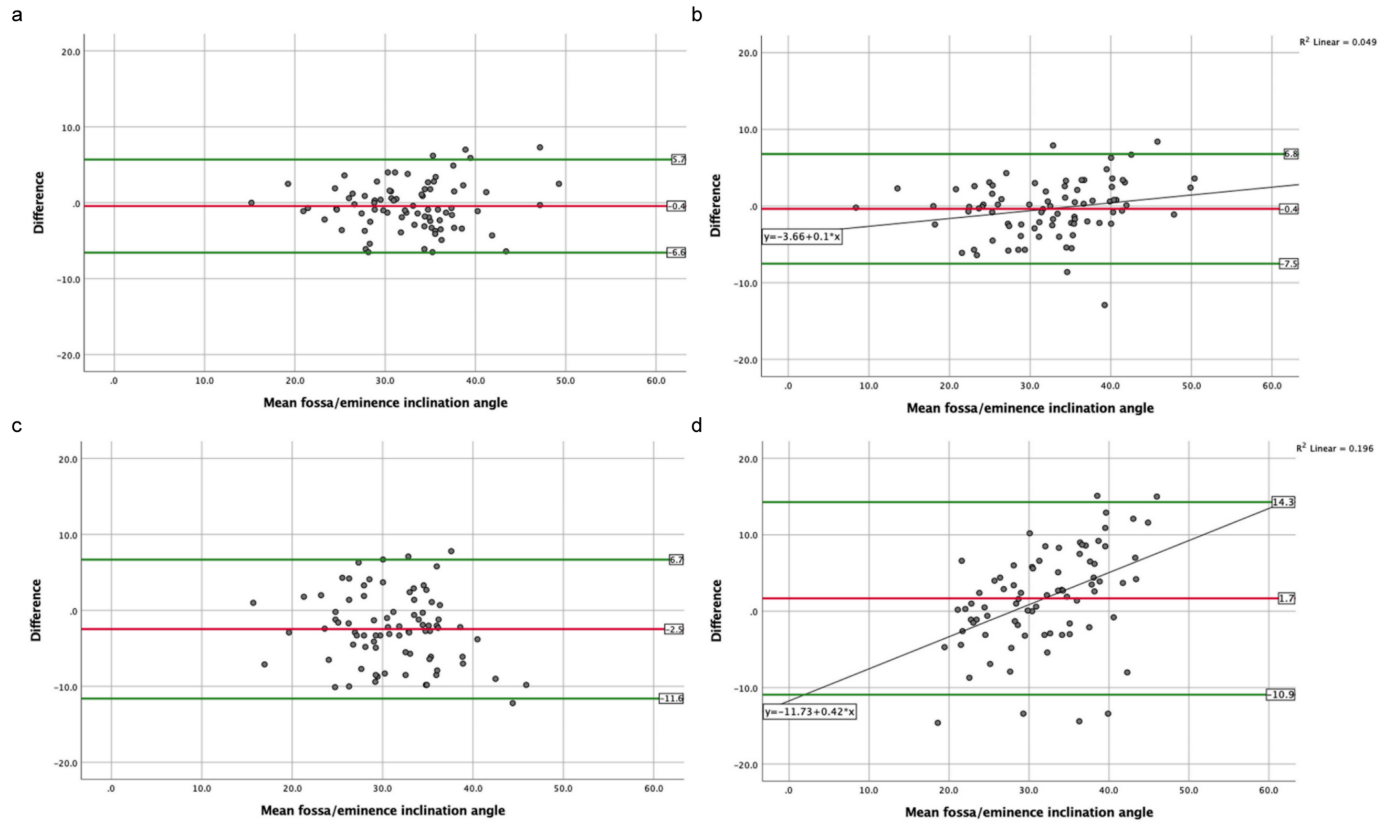

Supplement: Supplementary file 2 — Additional file 2. Examples of Bland–Altman plots without and with bias. [file 12903_2022_2701_MOESM2_ESM.docx]
